# Higher Order Aberrations following Scleral Buckling Surgery in Patients with Rhegmatogenous Retinal Detachment

**DOI:** 10.3390/healthcare9121643

**Published:** 2021-11-27

**Authors:** Chia-Yi Lee, Wei-Chi Wu, Ling Yeung, Hung-Chi Chen, Kuan-Jen Chen, Yen-Po Chen, Yih-Shiou Hwang, Chi-Chun Lai

**Affiliations:** 1 Department of Ophthalmology, Show Chwan Memorial Hospital, Changhua 50093, Taiwan; ao6u.3msn@hotmail.com; 2 Department of Ophthalmology, Linkou Chang Gung Memorial Hospital, Taoyuan 333, Taiwan; weichi666@gmail.com (W.-C.W.); cgr999@gmail.com (K.-J.C.); yihshiou.hwang@gmail.com (Y.-S.H.); 3 Department of Medicine, College of Medicine, Chang Gung University, Taoyuan 333, Taiwan; lingyeung@gmail.com (L.Y.); yenpo.chen@gmail.com (Y.-P.C.); chichun.lai@gmail.com (C.-C.L.); 4 Department of Ophthalmology, Keelung Chang Gung Memorial Hospital, Keelung 204, Taiwan; 5 Center for Tissue Engineering, Linkou Chang Gung Memorial Hospital, Taoyuan 333, Taiwan; 6 Department of Ophthalmology, Tucheng Municipal Hospital, New Taipei City 236, Taiwan

**Keywords:** higher order aberrations, scleral buckling, wavefront analysis, Zernike term, rhegmatogenous retinal detachment

## Abstract

We aim to evaluate magnitudes of higher order aberrations (HOAs) from 3rd–6th order after scleral buckling (SB) for rhegmatogenous retinal detachment (RRD). A retrospective cross-sectional study of 19 patients with RRD who received SB (six receiving encircling SB, thirteen receiving segmental SB) was conducted. A wavefront analysis for surveying HOAs and other ophthalmic parameters were collected. Data between operated and fellow eyes, and a subgroup analysis of operated eyes, were analyzed by the Mann–Whitney U test, while a generalized linear model was applied to evaluate the correlation of HOAs to best-corrected visual acuity (BCVA) and optical symptoms. BCVA in the operated eyes was significantly worse (LogMAR: 0.18 ± 0.23 versus 0.05 ± 0.07, *p* = 0.001). Tilt (0.32 ± 0.14 versus 0.13 ± 0.08, *p* = 0.004), defocus (1.78 ± 0.47 versus 1.05 ± 0.17, *p* = 0.019) and coma (0.43 ± 0.11 versus 0.27 ± 0.09, *p* = 0.016) were significantly increased after SB. All root mean square (RMS), including RMS-3, RMS-4 and total RMS, were higher in operated eyes (all *p* < 0.05). Regarding Zernike terms, a significant elevation of vertical coma in the operated eyes was found (*p* = 0.038). In addition, tilt (0.41 ± 0.10 versus 0.17 ± 0.12, *p* = 0.007), defocus (2.27 ± 0.58 versus 0.82 ± 0.39, *p* = 0.001) and coma (0.59 ± 0.17 versus 0.11 ± 0.10, *p* = 0.015) were higher in the segmental subgroup, whereas spherical aberration (SA) was higher in the encircling subgroup (0.22 ± 0.04 versus 0.40 ± 0.15, *p* = 0.024) and RMS-4 and total RMS were increased in the segmental subgroup (both *p* < 0.05). Besides, tilt was correlated to worse BCVA (*p* = 0.036), whereas all four HOAs were correlated to the presence of optical symptoms (all *p* < 0.05). In conclusion, SB may increase HOAs, which could be associated with unfavorable postoperative visual outcomes and subject symptoms.

## 1. Introduction

Scleral buckling (SB) is a common surgical treatments for rhegmatogenous retinal detachment (RRD), with a reattachment rate of higher than 80% after primary surgery [1,2,3]. However, the visual recovery is not always satisfactory because of refractive change after SB [4,5,6], which is related to global shape changes, with a subsequent change in the axial length [7,8] and corneal shape [9,10], and an anterior segment anatomy change [11]. On the other hand, it is not uncommon for patients to complain of a reduced quality of vision, even if the retina is reattached postoperatively and the refractive error is fully corrected by appropriate optical lenses [12]. 

By decomposing the ocular wavefronts into Zernike polynomials, aberrations other than defocus and astigmatism can be identified and assessed [13,14], which is an application of wavefront technology for evaluating subtle refractive errors. Higher order aberrations (HOAs) are sums of aberrations with higher than the second Zernike order that are measured by wavefront technology and may influence visual quality, with symptoms such as night myopia, diplopia, halos and glare [13,14]. Various keratoplasty procedures induce HOAs [15,16], with penetrating keratoplasty having the largest effect [15,16,17]. Other intraocular surgeries, including trabeculectomy and cataract surgery, also increase the HOAs, with a minor effect on the long-term visual quality [18,19].

In contrast to intraocular surgeries, extraocular procedures theoretically produce more HOAs, since they tend to change the corneal curvature [20,21,22]. Orthokeratology is an intervention that changes the corneal shape to correct myopia, and an increment of 3rd-order and 4th-order aberrations has been reported [23]. In addition, lateral rectus recession has been proven to increase HOAs, at least during the first month postoperatively [24], whereas a pterygium excision has been found to reduce the HOAs after a 1-year period postoperatively [25]. Laser in situ keratomileusus (LASIK) has been shown to change the HOAs with different patterns between myopic and hyperopic LASIK [26]. Previously, it has been elegantly demonstrated that SB increases the total amount of HOAs, which is more prominent in the segmental than the encircling procedures [27]. However, only the 3rd-order and 4th-order HOAs were analyzed in that study.

The aim of our study was to investigate the magnitudes of HOAs from 3rd-order to 6th-order in eyes undergoing segmental or encircling SB for RRD. Besides, the correlation between HOAs in patients receiving SB to their visual acuity and persistent optical symptoms was also analyzed.

## 2. Materials and Methods

### 2.1. Subjects

A retrospective cross-sectional study was conducted, and 19 eyes of 19 patients (eight men and eleven women) that were diagnosed with RRD and received SB with a follow up period for at least six months were included, because we think this follow up period is adequate for a person receiving ophthalmic surgery to achieve a stable general ocular condition. Nineteen contralateral eyes among these 19 patients without prominent ocular co-morbidities, including corneal, glaucomatous and retinal disorders, served as the control group. Exclusion criteria included (1) a history of any ocular surgery, such as primary vitrectomy, cataract surgery, refractive surgery, glaucoma surgery or corneal transplantation, and (2) ophthalmic disorders, except for a myopic or hyperopic astigmatism with less than 2.00 diopters (D) cylinder, which means that the range of astigmatism was from −2D to +2D cylinder for all of the participants in the current study. The reason to exclude patients with astigmatism with more than 2D cylinder is to prevent the significant influence of high astigmatism on the visual performance in patients whose vision is already damaged by RRD. The etiology of RRD and systemic disease of each participant prior to the SB procedure was collected from the medical document.

### 2.2. Surgical Procedures

The patients were categorized into two subgroups according to the types of SB, and six patients were treated with the encircling buckling procedure, whereas 13 patients received the segmental buckling procedure. The encircling procedure was performed if a RRD range over 180 degree of retina was found. All SB were performed using standard techniques [28], and by one experienced vitreoretinal surgeon (L.Y.) in an interval of three years. Briefly, the surgical procedure included cryopexy and meticulous localization of breaks using scleral depression, as well as binocular indirect ophthalmoscopy. A segmental or circumferential silicone sponge buckle (506 style) was then sutured with matrix 5–0 Dacron sutures. The bandings for the segmental method were inserted at multiple locations of eyeballs. In some patients, trans-scleral drainage of subretinal fluid was accomplished by cut-down drainage technique using a 25-gauge needle after the choroid was exposed by a 2.00 mm long scleral dissection. Sulfur hexafluoride (SF6), octafluoropropane (C3F8) or room air was injected into the vitreous cavity if necessary.

### 2.3. Ophthalmic Examinations

The HOAs were measured at least three months postoperatively by using Hartmann–Shack aberrometer (KR-1W, Topcon Corp., Oakland, NJ, USA) with natural pupil size, which ranged from 3 to 4 mm according to the medical records. The HOAs measurements were performed three times and the mean values were used for our analysis. The data were transferred to the form of orthogonal Zernike polynomials, and then the magnitudes of the coefficients were shown as root mean square (RMS), meaning the square root of arithmetic mean of the squares of values, and used to show the wavefront aberrations. RMSs of 3rd- and 4th-order HOAs (RMS-3 and RMS-4) were obtained and analyzed separately, while the total amount of RMS was also analyzed. The postoperative best-corrected visual acuity (BCVA) was obtained via Snellen chart at 6 meters, and then transformed into LogMAR form for analysis. In addition, the auto-keratorefractometer (KR-7000, Topcon, Yamagata, Japan), A-scan biometry (Echoscan US-800; Nidek, Tokyo, Japan) ultrasonic pachymetry (USP; Micropach model 200P, Sonomed, Inc., Lake Success, North Hempstead, NY, USA), Orbscan II scanning-slit corneal topography (Orbtek Inc., Salt Lake City, UT, USA) and Tono-Pen II XL (Medtronic, Jacksonville, FL, USA) were applied to evaluate the refractive errors, axial length, central corneal thickness (CCT), corneal curvature and intraocular pressure (IOP), respectively. In addition, the optical symptoms, including photopsia, photophobia, glare, halo and monocular diplopia, that persisted six months postoperatively were obtained and analyzed, regardless of whether the abovementioned optical symptoms were present or not according to the medical records in outpatient department after the SB surgery. Patients were asked if the five optical symptoms were present after the surgery, and only the optical symptoms occurred after the SB surgery was counted in the current study.

### 2.4. Statistical Analysis

All data were analyzed using SPSS 20 (SPSS Inc. Chicago, IL, USA). Firstly, the descriptive analysis with mean and standard deviation (SD) show the demography of the study population. We used the Shapiro–Wilk normality test to check whether the population is normally distributed, which yielded a value of 0.027; thus, we chose nonparametric exam for the subsequent analyses. Then, the Mann–Whitney U test was used to compare the BCVA presented in LogMAR, refractive errors, biometry data, IOP, HOAs, RMS and the Zernike terms of the 3rd HOAs, as well as spherical aberration (SA) between the operated eyes and the fellow eyes. For the subgroup analysis in the operated eyes, the difference in HOAs and RMS between the segmental and encircling subgroups was analyzed with Mann–Whitney U test again. Moreover, the generalized linear model was utilized to evaluate both the potential correlation of HOAs to the BCVA and the presence of any optical symptom via yielding the odds ratio (OR) and corresponding 95% confidence interval (CI). We adjusted the link function to inverse and logit form in the generalized linear model to calculate the OR for BCVA and optical symptom presence with proper variance. A *p* value less than 0.05 would be regarded as statistically significant.

## 3. Results

### Subject Characteristics

Nineteen patients with RRD were selected in the current study: eight men and eleven women, with an average age of 37.53 ± 14.36 years (ranging from 15 to 57). Five patients had RRD on their left eyes, whereas fourteen patients developed the disorder on their right eyes. The RRD occurred spontaneously in 18 patients, while one patient developed the disorder after a traumatic accident. Nevertheless, prominent corneal ectasia was not detected via topography, nor was corneal haze observed via a slit-lamp biomicroscope in the patient with traumatic RRD during the follow up interval. The other demography and site of segmental SB are shown in Table 1 and Table 2. The room air, SF6 and C3F8 were injected in six, nine and four patients, respectively. In addition, the most common postoperative optic symptom was photopsia, which occurred in six participants, while the numbers of the rest of the optical symptoms are listed in Table 1. No severe intraoperative and postoperative complications, such as IOP elevation or suprachoroidal hemorrhage, were observed in the patients.

The BCVA, refractive errors, biometry data, IOP and HOAs-related data between the operated and fellow eyes are shown in Table 3. The BCVA presented by logMAR were 0.18 ± 0.23 postoperatively in the operated eyes, which was significantly worse than the 0.05 ± 0.07 in the fellow eyes (*p* = 0.001), whereas the spherical error, cylinder error, axial length, CCT, corneal curvature and IOP between the two groups revealed no difference (all *p* > 0.05). Except for SA, the HOAs, including tilt (0.32 ± 0.14 versus 0.13 ± 0.08, *p* = 0.004), defocus (1.78 ± 0.47 versus 1.05 ± 0.17, *p* = 0.019) and coma (0.43 ± 0.11 versus 0.27 ± 0.09, *p* = 0.016), were statistically significantly higher in the operated eyes. Besides, the RMS-3 (0.17 ± 0.04 versus 0.12 ± 0.05, *p* = 0.008), RMS-4 (0.21 ± 0.07 versus 0.16 ± 0.06, *p* = 0.022) and total RMS (0.40 ± 0.11 versus 0.32 ± 0.09, *p* = 0.005) were also higher in the study group. Regarding the Zernike terms, only the vertical coma (Z_3_^−1^, 0.10 ± 0.06 versus 0.02 ± 0.02, *p* = 0.038) yielded significant elevation in the operated eyes.

The comparisons about HOAs-related data between the segmental and encircling subgroups are demonstrated in Table 4. The tilt (0.41 ± 0.10 versus 0.17 ± 0.12, *p* = 0.007), defocus (2.27 ± 0.58 versus 0.82 ± 0.39, *p* = 0.001) and coma (0.59 ± 0.17 versus 0.11 ± 0.10, *p* = 0.015) were significantly higher in the segmental subgroup, whereas SA was higher in the encircling subgroup (0.22 ± 0.04 versus 0.40 ± 0.15, *p* = 0.024). For RMS, the RMS-4 (0.30 ± 0.07 versus 0.04 ± 0.05, *p* = 0.006) and total RMS (0.52 ± 0.12 versus 0.23 ± 0.18, *p* = 0.032) were higher in the segmental subgroup, but RMS-3 demonstrated no significant difference (*p* = 0.061). 

**Figure 1:**
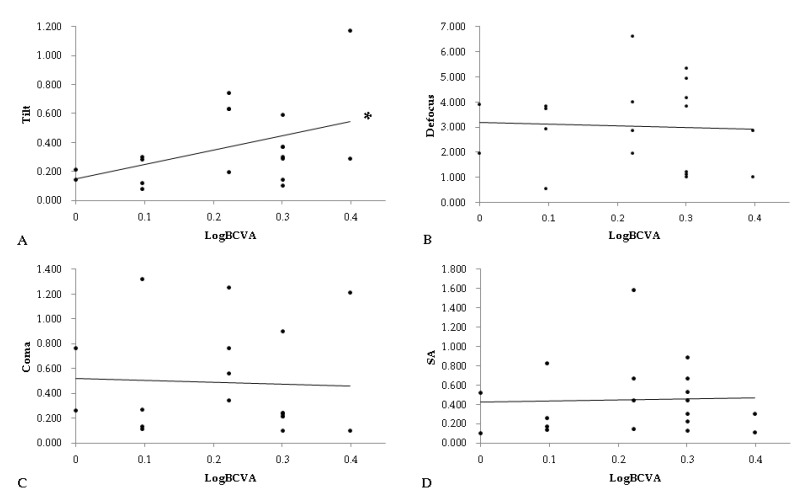
The correlation between best-corrected visual acuity and four types of higher order aberrations. (**A**): the correlation between BCVA and tilt, (**B**): the correlation between BCVA and defocus, (**C**): the correlation between BCVA and coma, (**D**): the correlation between BCVA and SA, BCVA: best-corrected visual acuity, SA: spherical aberration, *: denotes significant correlation between the two parameters.

The correlation and statistical significance between the BCVA and HOAs in the operated eyes are revealed in Table 5. A significantly positive correlation between the LogMAR value of BCVA and tilt (OR: 1.62, 95% CI: 1.54–1.79, *p* = 0.036) was found, whereas the correlations between BCVA and defocus, coma and SA yielded insignificant results (all *p* > 0.05). Furthermore, all of the four HOAs, including tilt (OR: 1.27, 95% CI: 1.03–1.54, *p* = 0.014), defocus (OR: 1.22, 95% CI: 1.05–1.47, *p* = 0.015), coma (OR: 1.30, 95% CI: 1.11–1.42, *p* = 0.008) and SA (OR: 1.34, 95% CI: 1.07–1.61, *p* = 0.003), were positively correlated with the presence of any types of optical symptoms six months after the SB procedure. The distributions between BCVA and tilt, defocus and coma, as well as SA, are shown in Figure 1.

## 4. Discussion

Visual acuity after SB surgery may be compromised [29] given the postoperative alternation of axial length and corneal curvature with concomitant myopic or hyperopic changes [22,30]. In addition, previous experience also revealed an increment of the third and fourth order RMSs after SB, whereas the vertical coma (Z_3_^−1^) became negative [27]. Similar to previous research [29,31], a decreased BCVA was found among the patients undergoing SB in the current study. Moreover, significantly higher values of HOAs and RMS were measured in the study group, except for the SA, which is similar to the results of a seminal paper [27], and may lead to a reduced postoperative BCVA and elevated optical symptoms.

Both lower order aberrations and HOAs were higher in the study group. Even if the SA was not different between the study and the control groups, the numerically mean value of SA in the operated eyes was still nearly two-folds higher than that in the fellow eyes (0.28 versus 0.16). Our results demonstrated that the procedure of SB indeed increased HOAs from the 1st-order to the 6th-order. In previous studies, SB would change the ocular structure and corneal surface, with a subsequent increased surface regularity index and surface asymmetry index [22,32,33]. Although the biometry indexes were similar between the operated and fellow eyes, we speculate that the SB may not only change the anterior segment structure but also may alter the whole globe contour and lead to a subsequent HOAs increment. On the other hand, the foveal contour change is less likely to be the reason for the elevated HOAs, since all of the patients in the current study owned a smooth foveal status according to the finding of the optical coherence tomography. Regarding the time interval from SB to HOAs collection, because we think the anterior segment condition, including the refractive status, could be stable three months after the SB surgery, we arranged for a wave front aberrometry exam after that time. For more details about the time interval, the shortest SB-to-HOAs period was 3.2 months and the longest SB-to-HOAs period was 11.9 months in the study population, and the SB-to-HOAs period was 6–8 months postoperatively in 11 of 19 patients. Consequently, the findings in the current study may present the HOAs status within one year after the SB surgery.

In a previous study [27], RMS-3 and RMS-4 were significantly increased three months postoperatively in the study group, whereas RMS-5 and RMS-6 were not recorded. In our study, not only were RMS-3 and RMS-4 significantly increased in the operated eyes, but the total RMS, including RMS-5 and RMS-6, was also increased, as shown in Table 3. The difference in the 5th- and 6th-order RMS indicates that SB may influence the whole aberrations rather than only the 1st- to 4th-orders. Since the total RMS reflects the absolute amount, as well as the quality of aberrations [34], the difference in total RMS at least supports our hypothesis that HOAs are elevated after SB. 

Okamoto et al. [27] revealed in patients receiving segmental SB that the values of vertical coma (Z_3_^−1^) at the upper quadrant became negative (*p* < 0.01). In contrast, our study revealed a generally more positive value in the patients after SB (*p* = 0.038). Even if we only considered the patients receiving segmental SB, such as the study population in the research written by Okamoto et al., the values of vertical coma in the current study were still positive (0.10 ± 0.06). The reason for this phenomenon probably resulted from the difference in the location and size of the banding segments or the measured method, while our location of buckles are shown in Table 2. The SA (Z_4_^0^) has a tendency to reduce without statistical significance, probably due to a compensation of the original aberrations by the presumed aberrations induced by SB itself. The other 4th-order aberrations demonstrated a tendency to increase.

If we separated the operated eyes into the segmental and encircling subgroups according to the type of SB they received, significant difference are shown regarding the aspects of tilt, defocus, coma, SA and RMS-4 via the Mann–Whitney U test. In addition, only the SA was higher in the encircling subgroup. The possible explanation is that SA is an aberration resulting from the difference in focus between the central ray and peripheral ray while the light passes a spherical surface. Accordingly, the encircling SB changes the gross sphere shape of eyeball more than the segmental SB due to the circular area of attachment and fixation, and thus may change the peripheral cornea more universally and lead to a higher SA. The total RMS has no significant difference, but the RMS-4 does, which may indicate that there are other HOAs or lower order aberrations that are higher in the encircling subgroup than the segmental subgroup and compensate for the 4th-order influence. However, this hypothesis needs further examination in order to be confirmed. Okamoto et al. have shown that the segmental procedure induces more HOAs than the encircling procedure [27], which is similar to the results of our study, where the segmental SB leads to a higher HOAs elevation in three of the four HOAs compared to the encircling counterparts.

The spherical errors, cylinder errors, axial length, CCT, corneal curvature and IOP did not show a significant difference in the current study, though SB is a compressive surgery and the axial length and IOP are supposed to be elevated according to previous experiences [30,35,36]. A possible explanation for the similar axial length between the two groups is the pre-existing high myopia status of the fellow eye (the lowest myopia in the fellow eye was a −4.5D sphere and the highest myopia was a −8.75D sphere) features, with a longer axial length originally. Besides, the mean value of AL in the current study is similar to a previous study that measured the axial length after SB [36], which may imply that the post-SB axial length is around this value. The numerically lower IOP in the operated eyes may be because the postoperative IOP after SB often declined to a normal range within one week [37], and this phenomenon may also indicate that patients with glaucoma are safe to receive SB, which is similar to previous findings [38].

There are still some limitations due to the retrospective nature of the current study. Firstly, the study population in the current study is too small, with only 38 eyes in 19 patients, which can lead to a significant statistical bias and diminish the credibility of the results from the current study. Besides, we did not record the preoperative data of the operated eyes; otherwise, we can compare the baseline data more accurately and may find some baseline difference, such as visual acuity or spherical errors. Fortunately, the HOAs, refraction status and visual performance are similar between contralateral eyes according to previous research [39,40]; thus, our findings may be accompanied with less disturbance from the missing data. Finally, the follow up period and intervals between SB surgery and ocular examinations were not identical between all patients due to the retrospective nature. Consequently, the patients were not followed in a uniform interval, as those in the prospective studies, and the HOAs amount at a specific postoperative time point could not be evaluated precisely.

## 5. Conclusions

In conclusion, SB may significantly enhance HOAs, which may correlate to reduced postoperative visual outcomes, especially if the tilt was elevated. Furthermore, the presence of any type of postoperative HOAs elevation may be correlated to the development of prolonged optical symptoms. The different types and extents in the enhancement of HOAs between the segmental and encircling groups suggest distinct mechanical effects between these two procedures. Further large-scale research with preoperative and postoperative data is warranted.

## Figures and Tables

**Table 1:** Basic demography of the whole population.

Demography	Value
Age (mean± SD, year)	37.53 ± 14.36
Sex (male: female, number)	8:11
Eye (right: left, number)	5:14
Co-morbidity (disease)	1 ^#^
Etiology of RRD	
Idiopathic	18
Traumatic	1
Gas injected (number)	
SF6	9
C3F8	4
Room air	6
Buckle type (number)	
Encircling	6
Segmental	13
Time from SB to HOAs exam (mean± SD, months)	7.65 ± 2.12
Optical symptom	
Photopsia	6
Photophobia	1

SD: standard deviation; RRD: rhegmatogenous retinal detachment; SF6: sulfur hexafluoride; C3F8: octafluoropropane; SB: scleral buckling; HOAs: higher order aberrations; ^#^ one patient was diagnosed with systemic lupus erythematous prior to scleral buckle procedure.

**Table 2:** Site of segmental scleral buckle implantation.

Patient No.	Eye	Site of RRD (o’clock)	Site of Sclerotomy (o’clock)	Quadrant
1	OD	12:00–02:30	02:00	Superonasal
2	OD	10:00–11:00	09:00	Superotemporal
3	OD	09:00–01:00	10:00	Superotemporal and superonasal
4	OD	02:00	01:00	Superonasal
5	OD	09:00–02:30	11:00	Superotemporal and superonasal
6	OS	03:00	03:00	Temporal
7	OD	11:00	10:00	Superotemporal
8	OS	02:00	02:00	Superotemporal
9	OD	09:30–02:30	11:00	Superotemporal and superonasal
10	OD	09:00	09:00	Temporal
11	OD	09:00–10:00	10:00	Superotemporal
12	OD	09:00–11:00	09:00	Superotemporal
13	OD	06:00	06:00	Inferotemporal and inferonasal

RRD: rhegmatogenous retinal detachment.

**Table 3:** Ocular indexes and higher order aberration data between the operated and fellow eyes.

Parameters (mean ± SD)	Operated Eye (*n* = 19)	Fellow Eye (*n* = 19)	*p* Value
Ocular indexes			
BCVA (LogMAR)	0.18 ± 0.23	0.05 ± 0.07	0.001 *
Spherical error (D)	−6.62 ± 1.84	−6.23 ± 1.30	0.825
Cylinder error (D)	−0.82 ± 0.34	−0.90 ± 0.45	0.792
Axial length (mm)	26.04 ± 1.98	26.01 ± 0.72	0.923
CCT (µm)	548.33 ± 21.67	554.86 ± 22.05	0.682
Corneal curvature (D)	43.92 ± 1.02	43.87 ± 0.37	0.904
IOP (mmHg)	14.54 ± 3.42	14.78 ± 3.68	0.960
HOAs			
Tilt	0.32 ± 0.14	0.13 ± 0.08	0.004 *
Defocus	1.78 ± 0.47	1.05 ± 0.17	0.019 *
Coma	0.43 ± 0.11	0.27 ± 0.09	0.016 *
SA	0.28 ± 0.05	0.16 ± 0.13	0.053
RMS			
RMS-3	0.17 ± 0.04	0.12 ± 0.05	0.008 *
RMS-4	0.21 ± 0.07	0.16 ± 0.06	0.022 *
RMS-total ^#^	0.40 ± 0.11	0.32 ± 0.09	0.005 *
Zernike terms			
Z (−3,3)	0.07 ± 0.06	0.04 ± 0.06	0.132
Z (−1,3)	0.10 ± 0.06	0.02 ± 0.02	0.038 *
Z (1,3)	0.01 ± 0.02	0.03 ± 0.02	0.075
Z (3,3)	0.06 ± 0.04	0.03 ± 0.03	0.094
Z (4,0)	0.17 ± 0.09	0.29 ± 0.14	0.345

SD: standard deviation; BCVA: best-corrected visual acuities; D: diopter; CCT: central corneal thickness; IOP: intraocular pressure; HOAs: higher order aberrations; SA: spherical aberration; RMS: root mean square; * denotes significant difference between operated and fellow eyes; ^#^ the total RMS include the 3rd HOAs, 4th HOAs, 5th HOAs, such as pentafoil, secondary trefoil, secondary coma and 6th HOAs, such as hexafoil, secondary tetrafoil, tertiary astigmatism, secondary spherical aberration.

**Table 4:** The difference in higher order aberrations data between the segmental and encircling subgroups.

Parameters (mean ± SD)	Segmental Subgroup (*n* = 13)	Encircling Subgroup (*n* = 6)	*p* Value
HOAs			
Tilt	0.41 ± 0.10	0.17 ± 0.12	0.007 *
Defocus	2.27 ± 0.58	0.82 ± 0.39	0.001 *
Coma	0.59 ± 0.17	0.11 ± 0.10	0.015 *
SA	0.22 ± 0.04	0.40 ± 0.15	0.024 *
RMS			
RMS-3	0.20 ± 0.05	0.13 ± 0.08	0.061
RMS-4	0.30 ± 0.07	0.04 ± 0.05	0.006 *
RMS-total	0.52 ± 0.12	0.23 ± 0.18	0.032 *

SD: standard deviation; HOAs: higher order aberrations; SA: spherical aberration; RMS: root mean square; * denotes significant difference between the segmental subgroup and encircling subgroup.

**Table 5:** The correlations of higher order aberrations to the best-corrected visual acuity and optical symptoms.

HOAs	OR	95% CI	*p* Value
BCVA (LogMAR)			
Tilt	1.62	1.54–1.79	0.036 *
Defocus	0.91	0.86–1.43	0.512
Coma	1.00	0.73–1.62	0.701
SA	1.07	0.83–1.41	0.733
Optical symptoms ^#^			
Tilt	1.27	1.03–1.54	0.014 *
Defocus	1.22	1.05–1.47	0.015 *
Coma	1.30	1.11–1.42	0.008 *
SA	1.34	1.07–1.61	0.003 *

HOAs: higher order aberrations; BCVA: best-corrected visual acuities; SA: spherical aberration; * denotes significant correlation to the corresponded higher order aberration; ^#^ include photopsia, photophobia, glare, halo, monocular diplopia.

## Data Availability

The data included in the current study is available upon reasonable request.
